# Unsaturated Dinitriles Formation Routes in Extraterrestrial Environments: A Combined Experimental and Theoretical Investigation of the Reaction between Cyano Radicals and Cyanoethene (C_2_H_3_CN)

**DOI:** 10.1021/acs.jpca.2c01802

**Published:** 2022-05-31

**Authors:** Demian Marchione, Luca Mancini, Pengxiao Liang, Gianmarco Vanuzzo, Fernando Pirani, Dimitrios Skouteris, Marzio Rosi, Piergiorgio Casavecchia, Nadia Balucani

**Affiliations:** † Dipartimento di Chimica, Biologia e Biotecnologie, Università degli Studi di Perugia, 06123 Perugia, Italy; ‡ MASTER TEC, 06123 Perugia, Italy; ¶ Dipartimento di Ingegneria Civile ed Ambientale, Università degli Studi di Perugia, 06125 Perugia, Italy

## Abstract

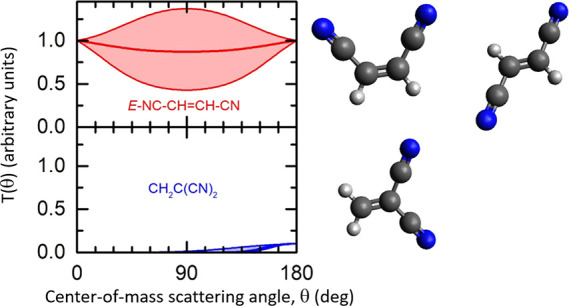

The reaction between cyano radicals (CN, X^2^Σ^+^) and cyanoethene (C_2_H_3_CN) has been investigated by a combined approach coupling crossed molecular beam (CMB) experiments with mass spectrometric detection and time-of-flight analysis at a collision energy of 44.6 kJ mol^–1^ and electronic structure calculations to determine the relevant potential energy surface. The experimental results can be interpreted by assuming the occurrence of a dominant reaction pathway leading to the two but-2-enedinitrile (1,2-dicyanothene) isomers (*E*- and *Z*-NC–CH=CH–CN) in a H-displacement channel and, to a much minor extent, to 1,1-dicyanoethene, CH_2_C(CN)_2_. In order to derive the product branching ratios under the conditions of the CMB experiments and at colder temperatures, including those relevant to Titan and to cold interstellar clouds, we have carried out RRKM statistical calculations using the relevant potential energy surface of the investigated reaction. We have also estimated the rate coefficient at very low temperatures by employing a semiempirical method for the treatment of long-range interactions. The reaction has been found to be barrierless and fast also under the low temperature conditions of cold interstellar clouds and the atmosphere of Titan. Astrophysical implications and comparison with literature data are also presented. On the basis of the present work, 1,2-dicyanothene and 1,1-dicyanothene are excellent candidates for the search of dinitriles in the interstellar medium.

## Introduction

Nitriles are a class of N-bearing organic molecules quite common in the interstellar medium (ISM), as they represent *ca*. 15% of the total detected species.^1^ Some of them are among the most complex organic molecules detected so far and are often highly unsaturated species (e.g., benzonitrile, cyanonaphthalene, vinylcyanoacetylene, cyanoacetyleneallene^2−5^). Nitriles have also been detected in the N-rich atmosphere of Titan^6,7^ and other small objects.^8^ Having an important prebiotic potential, their mechanisms of formation have been amply scrutinized in laboratory experiments and their possible role in the chemistry that triggered the emergence of life speculated (see, for instance, ref (9) and references therein). The detection of nitriles is favored by their significant dipole moments that produce intense rotational lines. In addition, their large abundance is certainly associated with the fact that their main precursor, the cyano radical, besides being ubiquitous and abundant in the ISM, is highly reactive with unsaturated organic molecules.^10^

Cyanoethene, C_2_H_3_CN, also commonly known as vinyl cyanide or acrylonitrile, with the IUPAC name being 2-propenenitrile, is the simplest olefinic nitrile and has quite large dipole moment components in the molecular plane of μ_*a*_ = 3.821 D and μ_*b*_ = 0.687 D.^11^ This has favored its detection in a variety of extraterrestrial environments through the identification of its transition lines in the microwave to THz frequency range^1^ with the support of laboratory data and calculations.^12−14^ The first detection was achieved in Sagittarius B2 in 1975,^15^ and more recently toward the hot molecular core Sgr B2(N)^16,17^ in Orion-KL,^18^ in the TMC-1 dark cloud,^19^ and in the L1544 proto-typical prestellar core,^20^ as well as toward IRAS 16293-2422^21^ and in the circumstellar envelope of the C-rich star IRC + 10216.^22^

Cyanoethene has also been detected in the upper atmosphere of Titan. Initially, its presence was inferred by the detection of the cation H_2_CCHCNH^+^ using the Ion and Neutral Mass Spectrometer (INMS)^23−26^ aboard the Cassini orbiter. However, the definitive proof for the presence of the neutral C_2_H_3_CN came from the detection of its rotational lines in the frequency range of 230–232 GHz analyzing the data from the Atacama Large Millimeter/submillimeter Array (ALMA) Science Archive.^27^ A follow-up study using higher sensitivity data, also taken from the ALMA archive, presented the very first spatially resolved map of the distribution of cyanoethene in Titan’s atmosphere.^28^ The presence of this species has attracted the attention of the astrobiology community because it appears to be the best candidate to form membrane-like structures in apolar solvents, such as the methane (CH_4_), suggesting a potential role in the methane/ethane lakes present on the surface of Titan.^29^

The main formation route of C_2_H_3_CN in interstellar objects and planetary atmospheres is known to be the gas-phase neutral–neutral reaction of cyano radicals (CN) with ethene (C_2_H_4_) via an H-displacement mechanism. The reaction has been found to be very fast also at the very low temperatures which are typical of interstellar clouds and Titan with a rate coefficient of the order of 10^–10^ cm^3^ mol^–1^ s^–1^ according to CRESU (Cinetique de Reaction en Ecoulement Supersonique Uniforme) experiments.^30^ This reaction was also explored by means of the crossed molecular beam (CMB) method with mass-spectrometric (MS) detection^31−35^ and characterized by electronic structure calculations of the potential energy surface (PES).^35,36^ The CMB-MS experiments demonstrated that the channel leading to the formation of cyanoethene and atomic hydrogen is the dominant pathway, even though at high collision energies the isocyanoethene isomer can be formed with a very small yield.^35^ Later on, the kinetics experiment by Gannon et al.^37^ confirmed that only the H-displacement channel is open at room temperature and at 195 K. The theoretical investigation of the reaction mechanism clearly demonstrated that the reaction is initiated by the CN attack to the π system of ethene leading to the addition of CN to one of the two equivalent C atoms followed by the elimination of an H atom.^32,35,36^ The addition can occur both on the C-side and on the N-side. However, the most probable destiny of the N-side addition intermediates is to isomerize to their cyano counterparts rather than forming isocyanoethene as the final molecular product.^32,35^ Only at high collision energies was there some evidence of the isocyanoethene formation visible.^34,35^

The CN radical is ubiquitous and largely abundant in the interstellar medium. In the upper atmosphere of Titan, it is formed by the photodissociation of hydrogen cyanide (HCN), the most abundant nitrile in that environment.^6,7^ Therefore, given the efficiency of the CN reaction with olefins, it is plausible that newly synthesized cyanoethene molecules could further react with another CN radical resulting into an even more complex CN-bearing species. In the case of the atmosphere of Titan^6,7^ the molar fraction of cyanoethene is (3.46 ± 0.51) × 10^–7^ at 1050 km as inferred using the INMS in closed-source neutral (CSN) mode and by fitting the INMS CSN signal using cracking patterns of multiple species.^25^ Interestingly, a higher molar fraction of about 10^–5^ at 1100 km was reported by Vuitton and co-workers,^23^ with this value being closer to the predicted abundances of recent models.^7,27^ This value has also been confirmed by the analysis of the ALMA data.^27^ The molar abundances for HCN (that would ultimately lead to CN by photodissociation) are at least 1 order of magnitude higher at similar altitudes (2.44 ± 0.10) × 10^–4^ at 1050 km and 2 × 10^–4^ at 1100 km).^7,23,25^ Photodissociation of HCN generating CN radicals is particularly efficient between 800 km and 1000 km of altitude^7^ and leads to a predicted abundance of ca. 10^–7^ at 1100 km of altitude according to recent models.^7^ Therefore, although most of the freshly produced CN will react with C_2_H_6_ (reforming HCN) or with the abundant C_2_H_4_ and C_2_H_2_, the reaction CN + C_2_H_3_CN can certainly occur and could possibly lead to more complex nitriles.

Analogously, it is interesting to note that in the molecular cloud TMC-1 (the cold cloud where many unsaturated nitriles have been detected) the measured column density of cyanoethene is 3 × 10^12^ cm^–2^ giving an abundance, with respect to hydrogen (10^22^ cm^–2^), of 3 × 10^–10^.^19^ The abundance of the CN radical in TMC-1,^38,39^ is also very large, being 7.4 × 10^–10^ (or as high as 2.9 × 10^–8^ as inferred by Crutcher and co-workers^40^), thus implying that the reaction between these two species could occur in cold molecular clouds as well.

Not much is known for the title reaction as well as for other gas-phase reactions involving cyanoethene.^41^ To the best of our knowledge, only a kinetic experiment on the reaction CN + C_2_H_3_CN is available with the rate coefficients derived at room temperature and above (298, 345, 425, and 528 K).^42^ The title reaction has been included in the photochemical models of Titan by Loison et al.^6^ and Vuitton et al.^7^ with a rate coefficient of 3.02 × 10^–11^e^+130/*T*^ which has been extrapolated from the kinetic data of Butterfield et al.^42^ by Hebrard et al.^43^ The only channel which was assumed to be open is H-displacement and a product with gross formula C_4_H_2_N_2_ is considered in both models. On the contrary, Hebrard et al.^43^ considered the products to end up into condensable macromolecules. If we assume that the reaction mechanism is similar to the one leading to the formation of cyanoethene, its reaction with cyano radicals should bring to the formation of dinitriles, either 1,1-dicyanoethene or 1,2-dicyanoethene. Dinitriles are an interesting category of compounds in prebiotic chemistry.^44^ Cyanogen (C_2_N_2_) and dicyanoacetylene (C_4_N_2_) have been detected in the atmosphere of Titan.^45,46^ There have been also some attempts to detect dinitriles in the interstellar medium after the recent detection of protonated cyanogen (NCCNH^+^)^47^ and isocyanogen (CNCN).^48^ However, the focus has been so far on saturated dinitriles, such as succinonitrile and glutaronitrile,^49−52^ because the class of dinitriles which is expected to be most abundant, dicyanopolyynes, lack a permanent electric dipole moment and cannot be detected through their rotational spectra. No clear detection has been achieved so far, but it should be stressed that there are no obvious formation routes of saturated dinitriles. Recent attempts to detect dicyanobenzene in TMC-1 for both ortho- and meta-isomers only provide upper limits for its abundance.^53^ Notably, olefinic nitriles have the advantage of having a dipole moment and a feasible formation route, as the present work is going to demonstrate with the firm identification of dicyanoethene as a primary product of the title reaction.

In this work, we report a combined experimental and theoretical study of the reaction of CN radicals with cyanoethene in order to cover the aforementioned lack of information. In particular, we have performed CMB experiments to derive the reaction mechanism, and we have derived the potential energy surface and performed RRKM estimates of the product branching ratio. We have also estimated the reaction rate coefficients at very low temperatures by relying on a semiempirical model and using as a guide the value of the rate coefficients measured at higher temperatures.

## Techniques and Methods

### Experimental Section

The CN(X^2^Σ^+^) + C_2_H_3_CN reaction was experimentally investigated using the CMB setup in Perugia. The apparatus and methodology is the same one reported in previous works to study the reaction dynamics of several atomic and radical species,^54−58^ and hence only a brief description of the setup and the details regarding the beams of cyanoethene and CN will be reported. The two reactants collide inside a high vacuum chamber with a base pressure of ca. 1.7 × 10^–7^ mbar rising to 2 × 10^–6^ mbar in operational conditions (beams on). The detection of the reaction products is carried out via a tunable electron impact ionizer followed by a quadrupole mass filter and a Daly type ion detector. The detector is housed inside a triply differentially pumped, ultrahigh vacuum (UHV) chamber (with a base pressure of 10^–10^ mbar) that can freely rotate around the reactive scattering center, in the plane of the two beams, to record the angular distribution of the scattered products. A time-of-flight (ToF) chopper wheel placed at the entrance of the detector allows us to measure the reactant and the product velocity distributions.

Cyanoethene (C_2_H_3_CN, Merck 99% purity, containing 35–45 ppm of monomethyl ether hydroquinone as inhibitor) was commercially available, and during the experiments, it was stored inside a glass vial, vacuum sealed, with an outlet to collect the vapor of C_2_H_3_CN. The C_2_H_3_CN reservoir was kept at a constant temperature of 292 K using a thermal bath of water–ethylene glycol in order to avoid temperature fluctuations. The supersonic beam of the nitrile molecule was obtained by expansion of 117 mbar (88 Torr) of the vapor through a 0.1 mm diameter stainless-steel (S.S.) nozzle, followed by a 0.8 mm S.S. skimmer and a further defining aperture. The resulting beam had a peak velocity of 621 m/s and a speed ratio of 3.8.

The CN beam was generated using a supersonic radiofrequency (RF) discharge beam source operating at 300 W,^34,59−61^ by expanding a gas mixture, with composition CO_2_ (0.75%) and N_2_(2.5%) in He at 90 mbar, through a 0.48 mm quartz nozzle followed by a 0.8 mm diameter boron nitride skimmer, and a further collimating aperture. Ions generated in the discharge are deflected using a 1800 V/cm electric field located between the skimmer and the defining slit. The CN radicals that are formed in the beams are ro-vibrationally excited, with a vibrational temperature of 6500 K, while the rotational distribution is bimodal, with two peaks around *N* = 6 and *N* = 39–44.^61^ However, for the similar systems CN + C_2_H_2_, C_2_H_4_, and CH_3_CCH,^32,61−64^ the reaction mechanisms derived in our CMB experiments employing the same CN beam are consistent with those obtained in another laboratory where CN is produced in a different way^65^ and with a different internal state distribution.^66^ In other words, the internal excitation of CN is not affecting the reaction mechanism and the triple C–N bond behaves as a spectator during the addition/elimination reaction. In the case of the present work, the resulting CN beam had a peak velocity of 2097 m/s and speed ratio of 5.5. Under these conditions the average collision energy (*E*_*c*_) of the experiment was 44.6 kJ mol^–1^.

Product laboratory angular distributions at selected mass-to-charge (*m*/*z*) ratios were acquired by modulating the C_2_H_3_CN beam at 160 Hz for background subtraction. Typical counting times were 100 s per angle. Product velocity distributions were measured using the pseudorandom chopping method^67^ at 6 μs channel^–1^, using a disk with four pseudorandom sequences of 127 open-closed slits. In all measurements, hard (70 eV) electron ionization (EI) was employed, because in this specific case there were no advantages in using soft ionization.^68,69^ In order to derive quantitative information on the reaction dynamics from the raw data, one has to move from the laboratory (LAB) framework to the center-of-mass (CM) frame and hence analyze the total CM product flux, *I*_*CM*_(θ,*E*_*T*_^′^)_*total*_. This can be conveniently factorized into the product angular, *T*(θ), and translational energy, *P*(*E*_*T*_^′^), distributions.^56,57^ Because of the finite experimental conditions (finite angular and velocity distributions of the two reactants and finite detector angular resolution), the best-fit CM functions are actually derived by a forward convolution fit of the total product LAB angular and ToF distributions at a certain *m*/*z* value as follows:
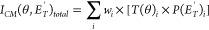
1with the parameter *w*_*i*_ representing the relative contribution of the apparent integral cross section of the *i*th channel, being a best-fit parameter.^57^

### Theoretical Section

The computational procedure at the base of our study is described in more detail elsewhere.^70,71^ Briefly, the lowest stationary points were calculated at the B3LYP level of theory^72,73^ with the correlation consistent valence polarized basis set aug-cc-pVTZ^74−76^ in order to explore the potential energy surface (PES) of the system CN(X^2^Σ^+^) + C_2_H_3_CN. Harmonic vibrational frequencies were computed at the same level of theory in order to check the nature of the stationary points, e.g., a minimum, if all the frequencies are real, and a saddle point if there is one, and only one, imaginary frequency. The assignment of the saddle points was performed using intrinsic reaction coordinate (IRC) calculations.^77,78^ In addition to this, single points (SP) CCSD(T)/aug-cc-pVTZ calculations were performed to derive more accurate energy values for all the stationary points.^79−81^ The zero-point energy correction, computed using the scaled harmonic vibrational frequencies at the B3LYP/aug-cc-pVTZ level, was then added to the B3LYP and the CCSD(T) energies of the SP calculations. Since the accuracy of our best computed values is around ±1 kcal mol^–1^, we rounded all the reported energies to 1 kJ mol^–1^. For all of our calculations we used the Gaussian 09 program suite,^82^ while Avogadro Version 1.2.0^83,84^ was our choice for the analysis of the vibrational frequencies.

Kinetics calculations were performed on the calculated PES using the code developed by our group to study similar reactive systems.^85−87^ The microcanonical reaction rate coefficient for each elementary step is calculated using the Rice–Ramsperger–Kassel–Marcus (RRKM) scheme. More precisely, the rate coefficient is given by the following expression:
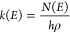
2Here *N*(*E*) is the sum of states in the transition state of energy *E*, ρ(*E*) is the reactant density of states at the energy *E*, and *h* is Planck’s constant. *N*(*E*) is obtained by integrating the relevant density of states up to energy *E*, assuming the rigid rotor/harmonic oscillator model.

Whenever possible, tunneling and quantum reflection have been taken into account by using the corresponding imaginary frequency of the transition state and calculating the tunneling probability for the relative Eckart barrier.

In previous work (see, for instance, refs (88−90)) we have used *capture theory* to determine the initial bimolecular association step, assuming that the entrance potential V(R) is of the form
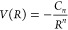
3where *R* is the distance between the two molecules, *C*_*n*_ is the interaction coefficient between the two species, and *n* = 6 in the case of neutral–neutral reaction or *n* = 4 if the process involves charged species. However, the results obtained with this approach largely overestimate the rate coefficient with respect to the experimental determination by Butterfield et al.^42^ (see below). Therefore, we resorted to a semiempirical model to evaluate the long-range interaction between the two reactants. The global intermolecular potential *V*_*TOT*_ can be defined as a combination of an electrostatic and a nonelectrostatic component:
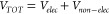
4

The electrostatic term *V*_*elec*_ is given by the equation
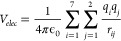
5where ϵ_0_ is the vacuum permittivity, *q*_*i*_ is the partial charge of each atom of the cyanoethene molecule, *q*_*j*_ is the partial charge of the two atoms of the CN radical, and *r*_*ij*_ is the distance between each pair *ij* of atoms in the interacting complex.

The evaluation of the nonelectrostatic component was done using the Improved Lennard-Jones (ILJ) functions. It has been demonstrated that ILJ gives a better reproduction of the intermolecular interaction over both short- and long-range distances with respect to the classical Lennard-Jones model.^91^ Accordingly, this component has been represented as pairwise additive *ij* contributions, each one evaluated at the proper *r*_*ij*_ distance:
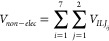
6

Each partial ILJ contribution is formulated as
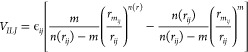
7where *m* = 6 must be chosen for neutral–neutral cases, while ϵ_*ij*_ and *r*_*mij*_ are, respectively, depth and location of the potential well, associated with the *ij* pair of interacting atoms.

The term *n*(*r*_*ij*_) shows a dependence on the distance *r*_*ij*_ defined as
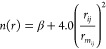
8

The parameter β, which relates to the hardness of the two interacting fragments, was set equal to 8, while ϵ_*ij*_ and *r*_*mij*_ depend on the value of the electronic polarizability,^92^ assigned at the interaction centers by the partition of the molecular polarizability values. After having calculated the potential according to (5) and (6), the corresponding *C*_*n*_ parameter is derived and used in the program that we usually employ to calculate the capture rate coefficient.

Differently with respect to what we have done in previous cases where a clear well is associated with the van der Waals complex lying between the long-range capture region and the first covalently bound intermediate,^88,93^ in this case, we have not treated the van der Waals complex as an intermediate in the RRKM calculations. This is because we have not been able to identify any transition state between the van der Waals and the first covalent intermediate. In other words, after the successful capture event, the reactive system proceeds naturally past the long-range complex into the intermediate region without the need to overcome a transition state, and there does not appear to be a significant submerged barrier. The very good comparison with the experimental data (see below) seems to sustain this view.

Once all the microcanonical rate constants were calculated, a Markov (stochastic) matrix^94^ is solved for all the intermediate and final product channels in the reaction. This Markov matrix is subsequently raised to a high enough power to achieve convergence, allowing us to derive the branching ratios (BRs) for all product channels in the desired temperature range.

## Results and Discussion

### Electronic Structure Calculations

All the possible reaction channels, along with their corresponding standard reaction enthalpies at the CCSD(T)/aug-cc-pVTZ level of calculations are listed in Table 1.

**Table 1:** List of Reaction Products and Their Standard Reaction Enthalpies

reaction			
		*ΔH*_0_^0^ (kJ mol^–1^)	label
CN + C_2_H_3_CN	→ H + *E*-NC–CH=CH–CN	–72	**1a**
	→ H + *Z*-NC–CH=CH–CN	–68	**1b**
	→ H + CH_2_=C(CN)_2_	–59	**1c**
	→ HCN + CH=CHCN	–50	**1d**
	→ NCCN + CH_2_CH	–21	**1e**
	→ H + *E*-NC–CH=CH–NC	16	**1f**
	→ H + *Z*-NC–CH=CH–NC	18	**1g**
	→ H + CH_2_=CCNNC	31	**1h**
	→ CNCN + CH_2_CH	79	**1i**
	→ H + H_2_CC(N)CCN	152	**1l**
	→ HCCN + CH_2_CN	159	**1m**
	→ H + NC–CH_2_C–CN	223	**1n**
	→ H + HCCHCNCN	291	**1o**
	→ CN + NC–CH_2_CH	354	**1p**

Only five channels are exothermic (namely, **1a**–**1e**) while all the others are endothermic (**1f**–**1p**). A further distinction among these reactive channels can be made based on whether the reactive pathways originate from the attack of the CN through the C-side or N-side.

Among the endothermic channels, **1i**–**1p** are not open under the conditions of our experiments because the collision energy is not high enough. For the same reason, they are not open under the low temperature conditions typical of both the atmosphere of Titan and interstellar clouds.

**Figure 1:**
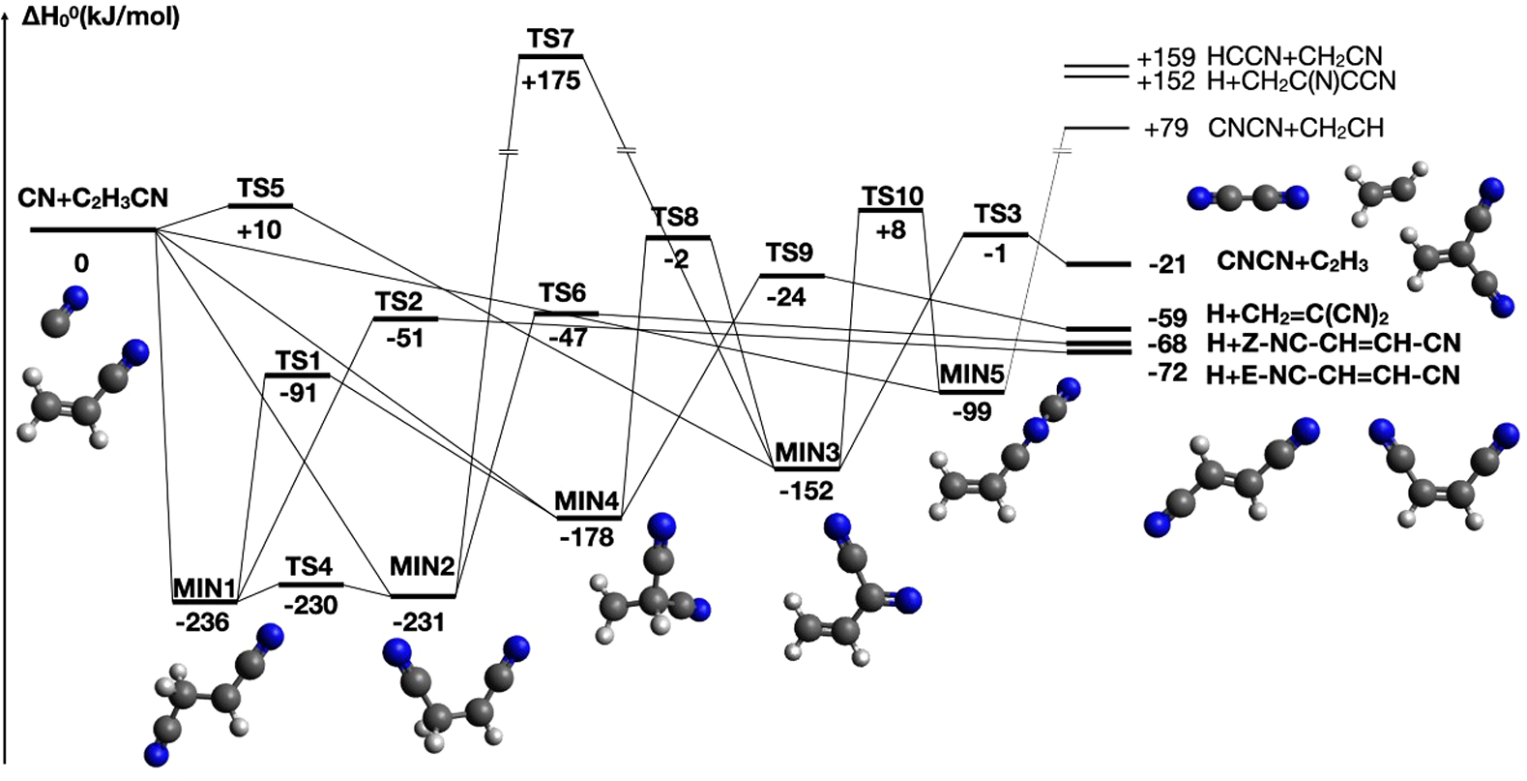
Schematic potential energy surface for the reaction CN(X^2^Σ^+^) + C_2_H_3_CN. Addition on the C-side of the CN radical.

**Figure 2:**
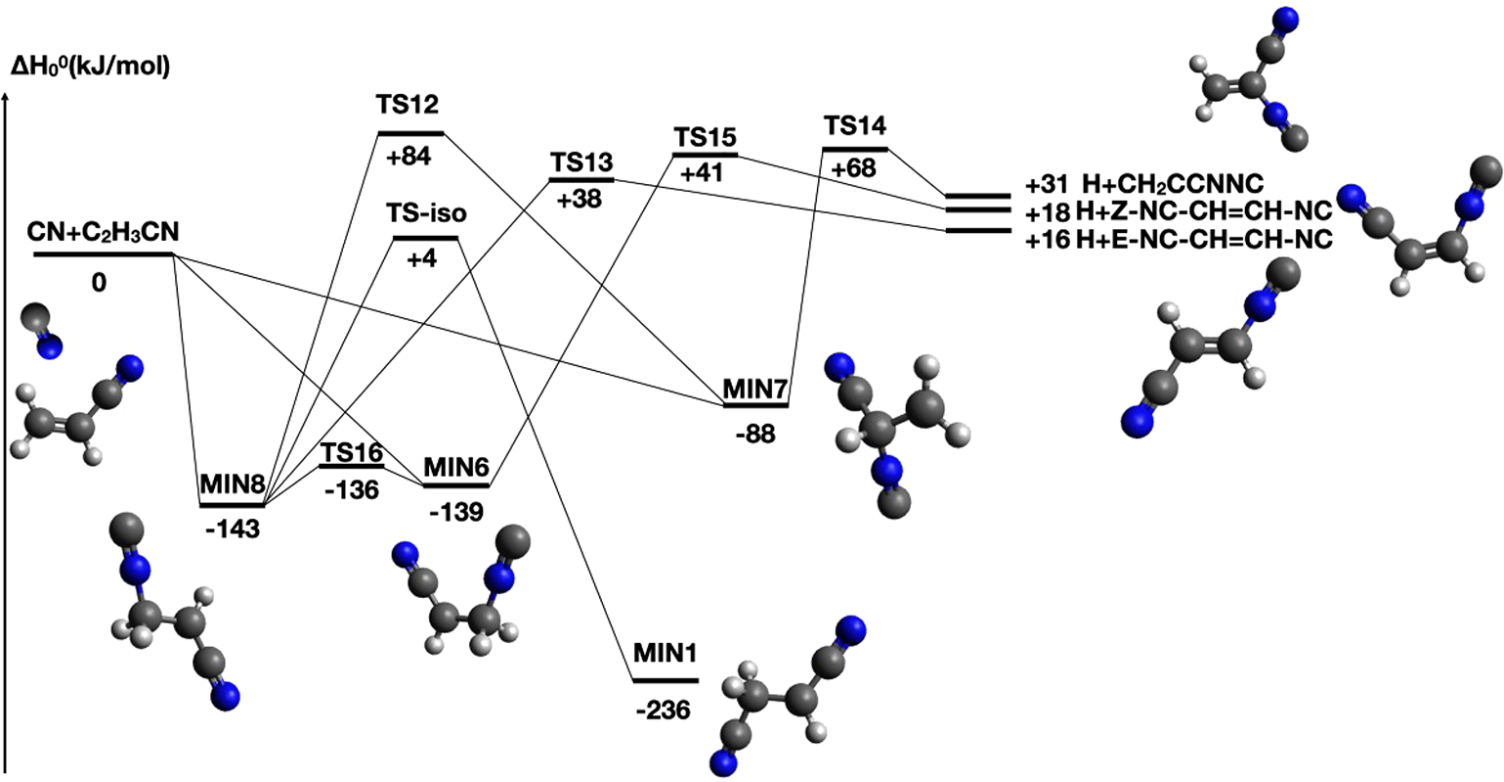
Schematic potential energy surface for the reaction CN(X^2^Σ^+^) + C_2_H_3_CN. Addition on the N-side of the CN radical.

A schematic representation of the C_4_H_3_N_2_ potential energy surface is reported in Figure 1 for the pathways starting with the CN addition to the π bond of ethene on the carbon side and in Figure 2 for the pathways starting with the CN addition on the nitrogen side. Table 2 lists the reaction enthalpies and the corresponding barrier heights (if present) for each step leading to the four exothermic channels **1a**–**1d**. Bond lengths and Cartesian coordinates for the minima, transition states, and products displayed in the PES are reported in the Supporting Information.

**Table 2:** Enthalpy Changes and Barrier Heights (kJ mol^–1^, 0 K) at the CCSD(T)/aug-cc-pVTZ Levels of Theory

reactive step	*ΔH*_0_^0^ (kJ mol^–1^)	barrier heights (kJ mol^–1^)
CN + C_2_H_3_CN → MIN1	–236	none
CN + C_2_H_3_CN → MIN2	–231	none
CN + C_2_H_3_CN → MIN3	–152	10
CN + C_2_H_3_CN → MIN4	–178	none
CN + C_2_H_3_CN → MIN5	–99	none
MIN1 → MIN2	4	5
MIN1 → MIN4	58	145
MIN1 → H + *E*-NC–CH=CH–CN	163	185
MIN1 → HCN + CH=CH–CN	186	223
MIN2 → MIN3	80	406
MIN2 → H + *Z*-NC–CH=CH–CN	163	184
MIN3 → MIN5	53	159
MIN3 → NCCN + CH_2_CH	131	151
MIN4 → MIN3	26	176
MIN4 → H + CH_2_=C(CN)_2_	119	154

The products of channel **1d** can be obtained either by a direct H-abstraction mechanism (characterized by an entrance barrier of 8 kJ mol^–1^) or by the decomposition of the MIN1 intermediate. In this case, however, the system needs to overcome a barrier of 223 kJ mol^–1^ because an H migration is required (the structure of the associated transition states TS-abs and TS-mig are shown in the Supporting Information).

Therefore, this channel is not expected to be competitive, either because of an entrance barrier (while the addition mechanism is barrierless) or because there are much easier dissociation pathways for MIN1.

According to our calculations, the interaction of the CN radical with the π bond of cyanoethene can lead, via a barrierless step, to the addition of CN to the terminal methylene carbon of the cyanoethene molecule leading to **MIN1** or its *E*-isomer **MIN2**. **MIN1** and **MIN2** easily interconvert to each other by overcoming a very small barrier associated with **TS4**. Alternatively, CN can also add to the C atom already bound to the nitrile group forming **MIN4** in a barrierless addition channel. Furthermore, two other initial interactions are possible: in one case the CN radical adds to the lone pair of the nitrogen atom of cyanoethene leading to **MIN5** (also this channel is barrierless). In the other case, the CN radical adds to the C atom of the nitrile group of cyanoethene forming the addition intermediate **MIN3**; this pathway, however, is characterized by an entrance barrier and this channel is not expected to be competitive with the barrierless approaches.

**MIN1** can evolve straight to the most exothermic products *E*-NC–CH=CH–CN + H (*E*-but-2-enedinitrile, **1a**) by overcoming **TS2**, or it can undergo isomerization to **MIN2** via a π rotation around the central carbon–carbon bond, having a very low barrier height (5 kJ mol^–1^) corresponding to **TS4**, or the CN radical can migrate to the C atom already bound to the pre-existing nitrile group, forming **MIN4** by bridging the two C atoms in **TS1**.

The **MIN2** intermediate can undergo H-elimination through an exit barrier located at −47 kJ mol^–1^ with respect to the energy of the reactants asymptote (**TS6**) leading to the second most exothermic product, *Z*-NC–CH=CH–CN (*Z*-but-2-enedinitrile, **1b**). **MIN2** can also isomerize to **MIN1** or to **MIN3** through a saddle point (**TS7**), which is, however, located 174 kJ mol^–1^ above the reactants asymptote. It follows that such a pathway becomes accessible only at very high energies and hence this route is expected to be negligible under our experimental conditions as well as in the extraterrestrial environments of interest.

The **MIN4** intermediate can lead to the 1,1-dicyanoethene (methylene–propanedinitrile), CH_2_C(CN)_2_, product by eliminating an H atom with an exit barrier located at 24 kJ mol^–1^ below the reactants asymptote (see saddle point **TS9** in Figure 1). In addition to that, **MIN4** can isomerize to **MIN1** by overcoming **TS1** located at −51 kJ mol^–1^ with respect to the reactants asymptote or isomerize to **MIN3** through the migration of the CN toward the C atom of the other nitrile group (the relative transition state is located at −2 kJ mol^–1^) .

**MIN3**, in turn, leads to the least exothermic pair of products, that is, NCCN + C_2_H_3_ (**1d**), through **TS3** following the C–C bond cleavage between the ethylene-like moiety and the cyanogen-like moiety. Moreover, it is also possible for the C–C bond in the cyanogen-like moiety in **MIN3** to break while a new bond forms between the N-side of one nitrile with the C-side of the other nitrile, leading to **MIN5**. This process, however, requires to overcome a transition state located at 8 kJ mol^–1^ above the reactants asymptote.

**MIN5**, which can also be formed directly from the reactants, could fragment into isocyanogen (CNCN) + C_2_H_3_ (**1h**) in a significantly endothermic channel. Therefore, this route does not occur either in the experimental conditions of our measurements or, even more so, in the colder environments of the interstellar medium and in Titan’s atmosphere.

In Figure 2 the possible intermediates, transition state and products formed when the CN addition takes place on N-side are shown. Also in this case, it is possible to have the barrierless formation of *Z*- and *E*- isomers (**MIN8** and **MIN6**) via the addition on the carbon of the methylene group or to **MIN7** via the addition on the N-side to the carbon bound to the nitrile group. **MIN8** can dissociate into the products *E*-NC–CH=CH–NC + H and **MIN6** into the products *Z*-NC–CH=CH–NC + H, while **MIN7** can evolve into 1-cyano-1-isocyanoethene. However, all the exit channels are endothermic and the transition states leading to the product formation are well above the reactants asymptote. **MIN8** can isomerize to **MIN6** or **MIN7** and *vice versa*. Finally, a transition state (**TS-iso**) located at +4 kJ mol^–1^ leads to the intermediate **MIN1** of the C-side addition PES. This is actually the lowest energy path in the N-side addition PES. On the basis of our calculations, we can conclude that the formation of isocyano molecular products is not competitive with the formation of their cyano isomers. Even though the addition on the N-side proceeds without a barrier, the most probable fate of the N-side-addition intermediates **MIN6**, **MIN7**, and **MIN8** will be to dissociate back to reactants or jump into the C-side addition PES via **TS-iso**.

### Crossed Beam Experiments

Reactive signal was observed at *m*/*z* = 77 and 78. The *m*/*z* = 77 distributions are superimposable to those recorded at *m*/*z* = 78, thus ruling out the possible occurrence of an H_2_-elimination channel. The signal-to-noise ratio (*S*/*N*) was similar for both *m*/*z* = 77 and *m*/*z* = 78, although slightly better for the latter. Because of the overlap with the strong elastic scattering signal associated with the reactants and their daughter ions, channel **1d** and **1e** could not be explored experimentally (the possible reactive scattering associated with the products at *m*/*z* = 52 would be completely submerged by the elastic scattering of −1 daughter ion of C_2_H_3_CN while for *m*/*z* = 27 the problem comes from interference of the CN beam through some mass leakage in the quadrupole mass filter caused the very intense signal at 26). The use of low energy ionizing electrons did not increase the signal-to-noise ratio for this system and, therefore, we have recorded product angular and TOF distributions at 70 eV.

**Figure 3:**
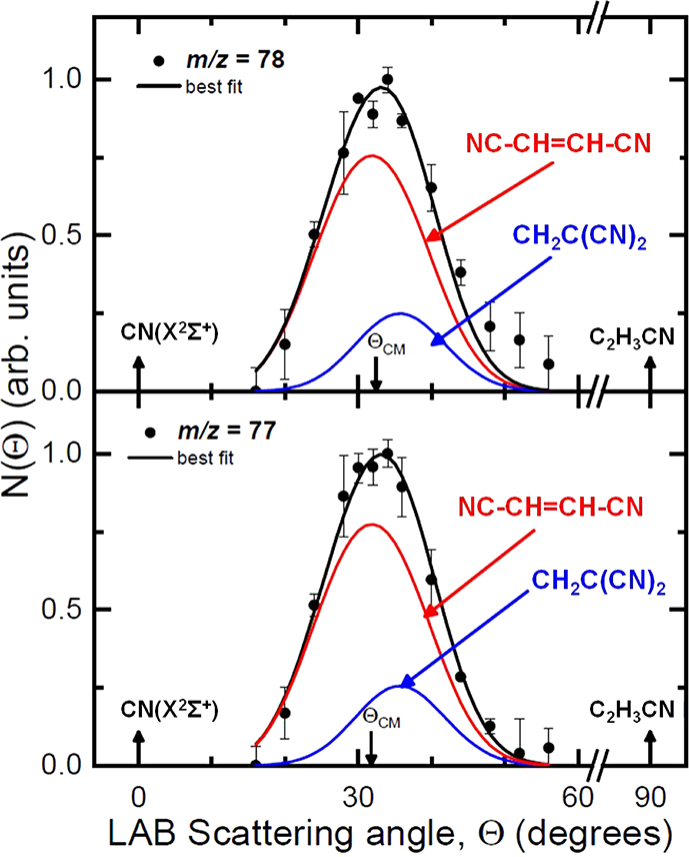
LAB angular distributions at *m*/*z* = 78 (top panel) and *m*/*z* = 77 (bottom panel) for the CN(X^2^Σ^+^) + C_2_H_3_CN reaction at *E*_*c*_ = 44.6 kJ mol^–1^. Error bars, when visible outside the dots, represent ±1 standard deviation from the mean value. The solid black curves are the distributions calculated with the best-fit CM functions. The curves in red and blue are the contributions to the signal from two reaction products of general formula C_4_H_2_N_2_.

**Figure 4:**
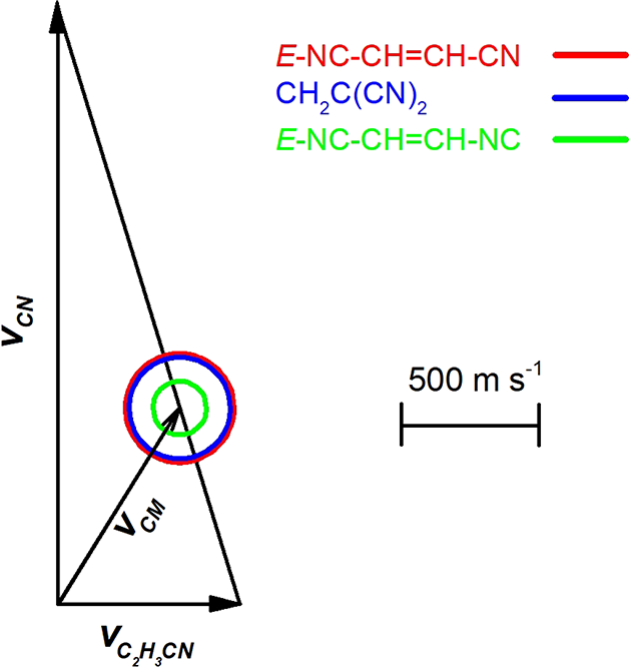
Newton (velocity vector) diagram with superimposed circles that delimit the CM speed (and angular range in the LAB frame) of the various indicated products of the CN(X^2^Σ^+^) + C_2_H_3_CN reaction at *E*_*c*_ = 44.6 kJ mol^–1^ (drawn by assuming that all the available energy is channeled into product translational energy when considering the reaction of CN in its ground rovibrational level (*v* = 0, *N* = 0)). The red, blue, and green continuous line are Newton circles for the H-displacement channels that lead to three isomers of formula *E*-NC–CH=CH–CN, CH_2_C(CN)_2_, and *E*-NC–CH=CH–NC, respectively. The *Z* isomer circles are not shown as they are indistinguishable from those of their *E* pairs in the compressed scale of the figure.

**Figure 5:**
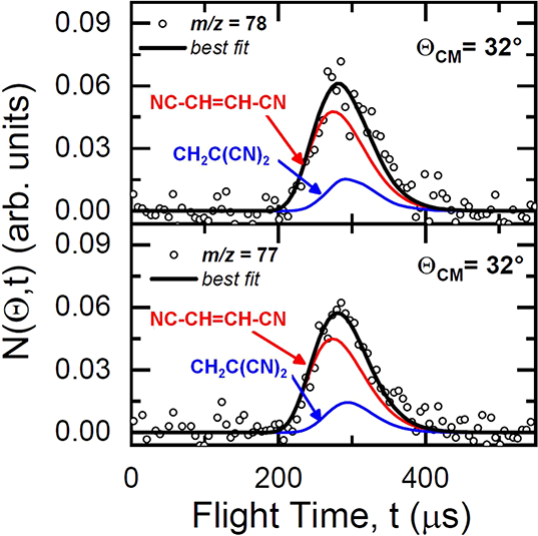
ToF distributions of the reaction products at *m*/*z* = 78 (top panel) and *m*/*z* = 77 (bottom panel) for the CN(X^2^Σ^+^) + C_2_H_3_CN reaction at *E*_*c*_ = 44.6 kJ mol^–1^ at the indicated LAB angles. The solid black curves are the distributions calculated with the best-fit CM functions. The curves in red and blue are the contributions to the signal from two reaction products of general formula C_4_H_2_N_2_.

Figure 3 shows the product laboratory (LAB) angular distribution obtained for the reactive signal at *m*/*z* = 78 (top panel) and *m*/*z* = 77 (bottom panel). The filled circles indicate the intensity averaged over the different scans while the error bars represent the ±1 standard deviation. Interestingly, each of the two LAB angular distributions are characterized by a relatively broad peak which is located slightly to the right of the center-of-mass angle (Θ_*CM*_ = 32°). The two distributions are essentially overlapping once the uncertainty is accounted for, thus suggesting that only one set of products is observed and that there are no contributions from possible H_2_-elimination channels. The relevant velocity vector (“Newton”) diagram is displayed in Figure 4. The Newton circles relative to the three possible isomers, *E*-NC–CH=CH–CN, *Z*-NC–CH=CH–CN, and CH_2_=C(CN)_2_ (channels **1a**–**1c**, continuous lines) and *E*-NC–CH=CH–NC, *Z*-NC–CH=CH–NC (channels **1m** and **1n**, dashed lines) are also shown, drawn under the assumption that all the available energy goes into product translational energy and considering only the reaction of CN (*v* = 0, *N* = 0). Unfortunately, given the kinematics of the experiment, the difference in the exothermicities of the channels **1a**–**1c** is too small, and the Newton circles for these three channels almost overlap. Since the Newton circles delimit the LAB angular range within which each specific isomer can be scattered, the angular ranges within which the three isomers of channels **1a**–**1c** are scattered are, therefore, very similar. On the contrary, the possible two isocyano–cyano isomers formed in the channels **1e**, **1f** are confined into much smaller angular ranges. Figure 5 presents the time-of-flight (ToF) spectra for the products detected at *m*/*z* = 78 (top panel) and *m*/*z* = 77 (bottom panel) at the LAB center-of-mass angle, Θ_*CM*_ = 32°.

**Figure 6:**
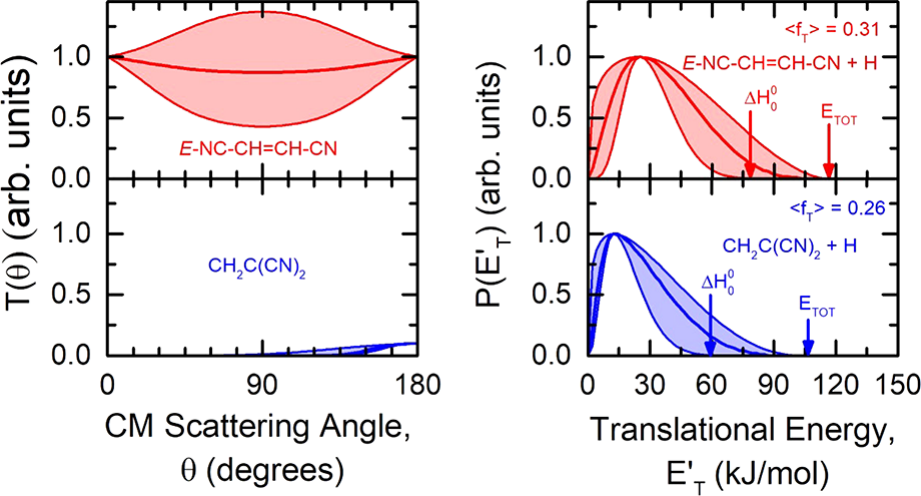
Best-fit CM angular and product translational energy distributions for the products of general formula C_4_H_2_N_2_ (red and blue lines) for the reaction CN(X^2^Σ^+^) + C_2_H_3_CN at *E*_*c*_ = −44.6 kJ mol^–1^. The red curves refer to the major reaction channel and the blue curves refer to the minor reactive channel. The total energy available to the three most stable products, (*E*_*TOT*_ = *E*_*c*_ – *ΔH*_0_^0^), and the corresponding exothermicty are indicated by a pair of arrows with their appropriate color match as displayed in Figure 4. The average fraction of energy released as product translational energy, ⟨*f*_*T*_⟩, is also indicated. *T*(θ) for the second contribution is normalized accordingly to the weight of the corresponding reaction channel.

The solid lines in both Figures 3 and 5 are the calculated curves using the forward convolution fitting procedure described in the experimental section. The best-fit condition was achieved by assuming two reaction channels, resulting in two sets of fitting functions. For each reaction channel, the differential cross-section in the CM frame was conveniently factorized into the product angular, *T*(θ), and translational energy, *P*(*E*_*T*_^′^), distributions, as shown in Figure 6, left and right panels, respectively. In particular, the arrows in the right panels of Figure 6 indicate the enthalpy changes associated with the three most exothermic products along with the relative total energy available when the collisional energy is taken into account.

From the analysis of our laboratory measurements, we can conclude that there are two channels contributing to the total integral cross-section among which one (channel 1) neatly dominates over the minor one (channel 2) with a best-fit relative ratio of *w*_2_/*w*_1_ = 0.1 ± 0.05.

In the case of channel 1, the CM angular distribution, *T*(θ), is backward-forward symmetric and nearly isotropic. Once the fit-uncertainty is taken into account, the *T*(θ) can either be more markedly polarized (lower bound) or with some preference for sideways scattering (upper bound). All these features point to a bimolecular reaction that proceeds through at least one stable intermediate (long-lived complex) with a lifetime, τ, longer than its rotational period, τ_*R*_. This is characteristic of the so-called *indirect mechanism* which is consistent with the PES having at least one deep potential well associated with an intermediate surviving for several rotational periods before dissociating into the final products and, hence, resulting in losing memory of the original direction of the reactants. Moreover, the *P*(*E*_*T*_^′^) of the dominant channel peaks at ca. 25 kJ mol^–1^, with an average product translational energy, defined as , of 36.0 kJ mol^–1^. This leads to a fraction of translational excitation, , of 0.31 with respect to the total available energy (*E*_*TOT*_ = *E*_*c*_ – *ΔH*_0_^0^ considering the reaction of CN in its ground rovibrational level). It follows that the final product is formed with significant rotational and vibrational excitation (counting as the remaining 69% of the total available energy).

In the case of channel 2, the best-fit CM angular distribution, *T*(θ), displays a strongly marked preference toward backward scattering which is a clear indication of a *direct, rebound mechanism* dominated by small impact parameters. These features can be interpreted by invoking the presence of an alternative minor mechanism that can be associated with the formation of 1,1-dicyanoethene if we assume that the attack of the CN radical on the C atom that is already bound to a nitrile group occurs only through a small acceptance cone. By contrast, the indirect mechanism of the dominant channel has a larger acceptance cone, and hence larger impact parameters, which is linked to the CN interaction with the electron density of the π orbital. The LAB distributions can also be simulated by using a single component fit. In this case, the global *T*(θ) has a clear backward propensity. A single component fit, however, does not allow us to evaluate the extent of the minor contribution, while the interpretation of the scattering data remains unchanged. The associated *P*(*E*_*T*_^′^) to the second minor reaction channel peaks at ca. 12 kJ mol^–1^, and it has an average product translational energy of 26.7 kJ mol^–1^. This means that the minor product is formed with a noticeably high degree of internal excitation (74%), while having a fraction of translational excitation of 0.26 with respect to the total available energy. This finding is consistent with the minor product being slower with respect to the major one, as it can be observed by the corresponding contributions to the ToF spectra in Figure 4.

### Kinetics and RRKM Estimates

**Figure 7:**
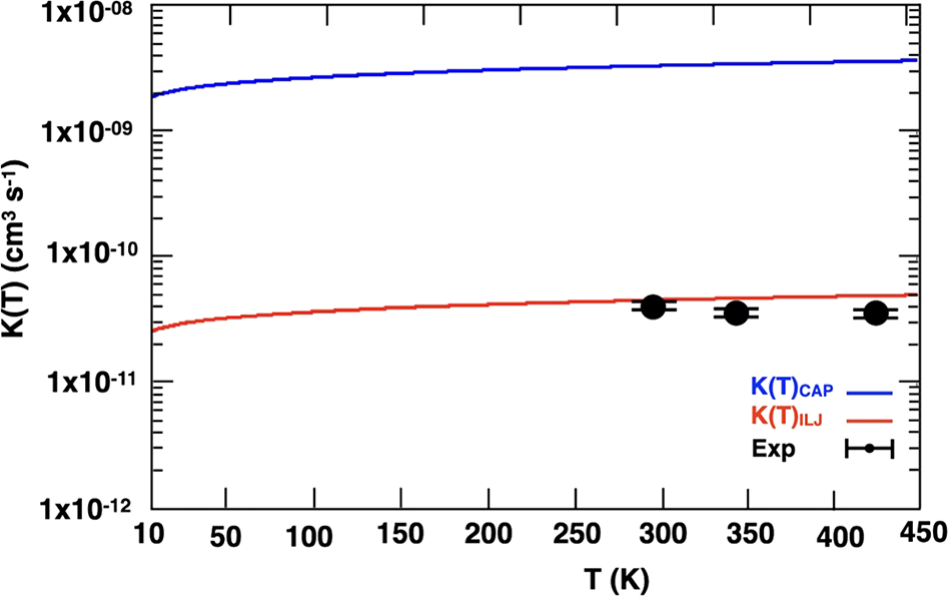
Rate coefficient as a function of the temperature. The experimental determinations at three different values of temperature by Butterfield et al.^42^ are also shown.

The global rate coefficient has been calculated with the method described in the theoretical section. The rate coefficients obtained with the traditional capture theory approach and with the semiempirical method illustrated in the “Theoretical Section” are reported in Figure 7. The experimental determinations at the temperature of 298, 345, and 425 K by Butterfield et al.^42^ are also shown in the figure (the kinetics calculations were carried out for temperature below 500 K). As is clearly visible, the sempiempirical method allows us to reproduce much better the experimental determination because the long-range region leading to the addition of CN to the π bond of cyanoethene is much better described.

The RRKM analysis was performed only by considering the partial PES calculated at the CCSD(T) level reported in Figure 1 accounting for the four exothermic reactive channels being, in order of increasing importance, those leading to *E*-but-2-enedinitrile (**1a**), *Z*-but-2-enedinitrile (**1b**), 1,1-dicyanoethene (**1c**), and cyanogen (**1d**). The contribution of all the other channels is indeed negligible, because those channels are characterized by the presence of high energy transition states and products. Notably, as already mentioned, the N-side addition mechanism does not lead to the formation of isocyano isomers, but rather, given the energy of the involved transition states, the addition intermediates **MIN6**, **MIN7** and **MIN8** will dissociate back to reactants or, via prior isomerization to **MIN8**, will access the C-addition PES via **TS-iso**.

The rate coefficients calculated for the four channels (**1a**–**1c**) as a function of the temperature have then been fitted using a modified Arrhenius law
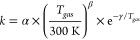
9which implies a linear variation of the activation energy with the temperature
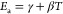
The best-fit resulting coefficients valid in the T range between 10 and 300 K are reported in Table 3 along with the calculated rate constants at selected temperatures representative of interstellar cold clouds, Titan’s surface, and Titan’s atmosphere.

**Table 3:** Rate Coefficients to Be Employed in Astrochemical and Photochemical Models

channel	α (cm^3^ s^–1^)	β (cm^3^ s^–1^)	*T* range (K)	*k*(10 K) (cm^3^ s^–1^)	*k*(94 K) (cm^3^ s^–1^)	*k*(175 K) (cm^3^ s^–1^)
**1a**	2.89 × 10^–11^	0.16	10–50	1.66 × 10^–11^	2.41 × 10^–11^	2.68 × 10^–11^
	2.94 × 10^–11^	0.18	51–150			
	2.95 × 10^–11^	0.18	151–300			
**1b**	1.57 × 10^–11^	0.16	10–50	8.69 × 10^–12^	1.29 × 10^–11^	1.49 × 10^–11^
	1.66 × 10^–11^	0.25	51–150			
	1.61 × 10^–11^	0.36	151–300			
**1c**	9.70 × 10^–14^	0.32	10–50	3.74 × 10^–14^	7.29 × 10^–14^	1.11 × 10^–13^
	1.44 × 10^–13^	0.89	51–150			
	1.08 × 10^–13^	1.89	151–300			
**1e**	3.82 × 10^–16^	1.82	10–50	3.67 × 10^–18^	8.82 × 10^–17^	6.97 × 10^–16^
	3.10 × 10^–15^	4.37	51–150			
	2.60 × 10^–15^	5.43	151–300			

These values have to be compared with those suggested by Hebrard et al.^43^ after the high *T* measurements by Butterfield et al.^42^ (the extrapolation by Hebrard is also implemented in the KIDA database^95^), namely 5.38 × 10^–11^ cm^3^ s^–1^ at 175 K and 8.84 × 10^–11^ cm^3^ s^–1^ at 94 K (to be compared with our global values of 4.17 × 10^–11^ and 3.70 × 10^–11^ cm^3^ s^–1^). The extrapolation at 10 K is outside the recommended range because, based on the work of Hébrard et al.,^96^ the uncertainty on the rate constant is 50% at 144 K, escalates quickly to 100% already at 102 K and keeps increasing at lower temperatures making any estimate at extremely cold temperatures not reliable. Our global value at 10 K is 2.53 × 10^–11^ cm^3^ s^–1^. Our determination is more accurate because, rather than being obtained by extrapolating the experimental values derived at much higher temperatures, it is based on dedicated electronic structure calculations and a long-range semiempirical potential that are able to reproduce the experimental high *T* values. This is well exemplified by the value at 10 K which becomes unphysically large in the extrapolation by Hebrard et al., while we actually derived a value which is somewhat smaller that those at 94 and 175 K. We note that the presence of a small submerged barrier (so small that it escaped our search) could reduce further the rate coefficients at very low *T*. Only an experimental determination at very low *T* could clarify this issue once and for all.

The branching ratios (BRs) at the aforementioned temperatures and at room temperature (300 K), and under experimental conditions, are listed in Table 4. The contribution from the **1e** channel is expected to be negligible at the investigated temperatures.

**Table 4:** Branching Ratios

	branching ratios				
channel	10 K	94 K	175 K	300 K	*E*_*c*_ = 44.6 kJ mol^–1^.
**1a**	0.65	0.65	0.64	0.63	0.60
**1b**	0.34	0.35	0.35	0.37	0.39
**1c**	1.5 × 10^–3^	2.0 × 10^–3^	2.6 × 10^–3^	4.2 × 10^–3^	0.01
**1e**	∼0	2.4 × 10^–6^	1.7 × 10^–5^	1.5 × 10^–4^	1.6 × 10^–3^

It is rather straightforward to note that the channel leading to *E*-but-2-enedinitrile (**1a**) is remarkably the dominant one accounting for 60% of the global reactive flux at the collision energy of our experiment and up to 65% at low temperatures. The second most relevant channel is the one leading to the second least stable isomer *Z*-but-2-enedinitrile (**1b**), counting up to 39% of the total yield. The channel ending in the formation of 1,1-dicyanoethene (**1c**) becomes somewhat appreciable only at the collision energy of our experiment counting for 1%, while it is negligible as the temperature decreases. The BRs of the remaining channel **1d** are nearly zero in the whole temperature range investigated.

## Discussion

The CMB data clearly suggest the occurrence of two different channels with two different reaction mechanisms that we have indicated with channels **1** and **2**. The CM functions associated with the major channel succeed in reproducing most of the characteristics of the laboratory angular distributions and ToF spectra at both mass-to-charge ratios for which it has been possible to record scattering signal (Figure 3 and Figure 5). The second channel accounts only for ca. 10% of the total cross section and is associated with the small propensity observed for backward scattering. For this reason, it makes sense to associate it to the formation of the 1,1-dicyanoethene isomer in the assumption that this product is formed only when the CN radical approaches cyanoethene on the side of the C atom that is already bound to a nitrile group with a small acceptance cone. All the other directions will instead inevitably lead to interaction with the π bond and to the more favorable addition on the C atom of the methylene group. As mentioned above, a single component fit with a global *T*(θ) with a clear backward propensity returns a fit that is nearly as good as the two-components best-fit. The interpretation of the scattering data, however, does not change because of the deep wells associated with **MIN1** and **MIN2**. We expect, indeed, that the formation of those strongly bound intermediates will generate either a backward-forward symmetric *T*(θ) or a best-fit *T*(θ) with some propensity for forward scattering if the lifetimes of the intermediates are comparable to, or smaller than, their rotational periods. RRKM estimates performed at the collision energy of our experiments indicates that only ca. 1% of channel **1c** contributes to the global reaction, but our RRKM calculations start from **MIN1** that rapidly equilibrates with **MIN2**. According to the interpretation of our scattering data, instead, the yield of the channel (**1c**) is associated with a direct mechanism that cannot be represented in a statistical approach, it being rather a dynamical effect.

Concerning the possible role of the internal excitation of CN on the scattering properties, we note that previous studies^34,35^ have already clarified the spectator role of the vibrational excitation of CN for similar reactions and justified this conclusion by noting that (i) the CN vibration does not affect the bond that is going to break, (ii) and the vibrational excitation of CN does not appear to be converted in translational energy (observed here as well), and therefore, it is retained as such in the final molecular products. However, the increased total energy available to the reactive system might reduce the lifetime of the involved intermediates.

The rotational excitation of CN could also have an effect on the reactive event as it was previously observed also for the CN + C_2_H_4_ reaction^34,97^ where a possible effect of CN rotational excitation in enhancing the CH_2_CHNC channel over the favored CH_2_CHCN one was suggested.

The results obtained in this study can be compared with the data reported in the literature for similar systems. In particular, we focused our attention on the reaction CN(X^2^Σ^+^) + C_2_H_4_ because cyanoethene can be regarded as an ethene molecule (C_2_H_4_) functionalized with a nitrile group in place of one of the four hydrogen atoms.^32,33^ Such comparison can be particularly appropriate when carried out with the work of Leonori et al.^34^ given the fact that the CN(X^2^Σ^+^) + C_2_H_4_ reaction was investigated using the same CMB apparatus described in the present work, and at a very similar collision energy (*E*_*c*_ = 42.7 kJ mol^–1^). For the analogous comparison of the PES, we can refer to the work of Balucani et al. instead.^31^ Starting from the experiments, the angular distributions for the two reactive systems share some common features. For instance, the maximum is located slightly at the right of the CM angle and the domains (LAB scattering angles) span a comparable range of values. Moreover, for both systems, two components are necessary to fit the data, with the minor channel being remarkably biased toward backward scattering in both cases.

The main product channel of the CN + C_2_H_4_ reaction displays a clear preference for the forward hemisphere which is consistent with the formation of an intermediate having a lifetime that is comparable to its rotational period (osculating complex). At lower collision energies, *T*(θ) was instead backward–forward symmetric with some propensity for sideways scattering^31^ In the title reaction, on the other hand, the presence of the CN group in the molecular skeleton of the reactant results in an increased number of degrees of freedom among which to distribute the large amount of energy liberated by the formation of the bound intermediates. This translates to an extended lifetime (τ) of the bound intermediate that will survive for longer than several rotations before dissociating into the final products and, in other words, in the appearance of a long-lived complex, instead of an osculating complex, governing the indirect reaction mechanisms.

In this regard, it is worthwhile highlighting that these two systems share many similarities, but with C_2_H_3_CN the symmetry of ethene has been lost. For both cases, the reactive event commences with a barrierless process producing intermediates at a nearly identical relative energy with respect to their reactants (−236 and −232 kJ mol^–1^). These minima evolve into the final products by overcoming an energy barrier of 185 and 153 kJ mol^–1^ for CN + C_2_H_3_CN and CN + C_2_H_4_, respectively. The presence of a higher exit barrier for one system with respect to the other suggests a faster rate constant for CN + C_2_H_4_ than for CN + C_2_H_3_CN. This also helps to increase the lifetime of the addition intermediate.

The similarities and differences in the PES of CN + C_2_H_4_ versus CN + C_2_H_3_CN, do not affect only the reaction dynamics, but more importantly, they have a direct impact on the reaction kinetics as well. This was demonstrated experimentally in the temperature range of 297–528 K, by the work of Butterfield et al.,^42^ reporting a reduction of the rate of the CN radical addition due to the electron-withdrawing effect of the cyano group. In contrast, the authors also observed the opposite effect, which is the enhancement of the rate constant, when the H atom on C_2_H_4_ was replaced with an electron donor group such as the methyl in the propene molecule (CH_2_CHCH_3_). In addition to that, it should be noted that for the reaction CN + ethene, also the N-side addition pathways could contribute to the overall reactive flux, because the transition state for the isomerization to the C-side addition PES is below the reactants asymptote, while for the title reaction it is slightly above.

**Figure 8:**
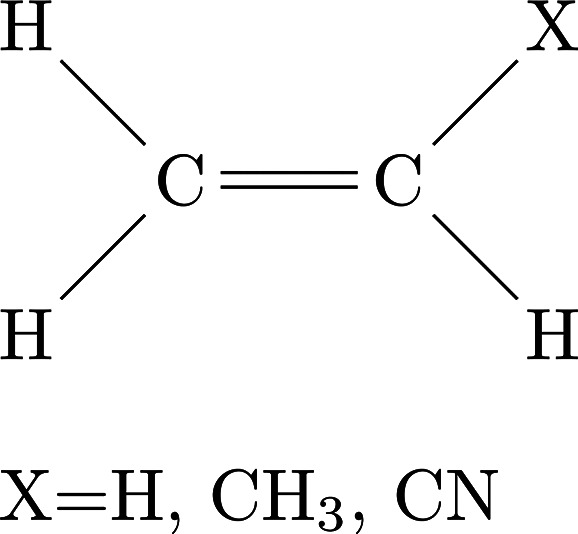
Schematic summarizing the structural similarities between ethylene, propene, and vinyl cyanide.

Figure 8 displays the common H_2_CC-backbone in ethylene, propene, and vinyl cyanide.

Table 5 lists some of the experimentally determined or extrapolated rate constants available in the literature for these three systems, CN + C_2_H_4_, CN + CH_2_CHCH_3_, and CN + C_2_H_3_CN, at three temperatures: around room temperature (RT), at 175 K, and at 25 K.

**Table 5:** Rate Constants for CN + C_2_H_4_, CN + H_2_CCHCH_3_, and CN + H_2_CCHCN

CN + H_2_CCHX	rate constant (cm^3^ s^–1^)	temperature (K)	ref
X = H	(2.5 ± 0.3) × 10^–10^	295	Sims et al.^30^
X = H	(2.6 ± 0.1) × 10^–10^	297	Yang et al.^98^
X = CH_3_	(2.87 ± 0.04) × 10^–10^	297	Butterfield et al.^42^
X = CH_3_	(3.03 ± 0.31) × 10^–10^	298	Morales et al.^99^
X = CN	(4.23 ± 0.08) × 10^–11^	297	Butterfield et al.^42^
X = H	(3.2 ± 0.5) × 10^–10^	175	Sims et al.^30^
X = CH_3_	((3.2 ± 0.9) × 10^–10^)^a^	175	Morales et al.^99^
X = CN	n/a	175	n/a
X = H	(4.4 ± 0.2) × 10^–10^	25	Sims et al.^30^
X = CH_3_	(3.7 ± 0.1) × 10^–10^	25	Morales et al.^99^
X = CN	n/a	25	n/a

a The error on the rate constant was estimated by propagating the error using the fit uncertainties provided by Morales and co-workers.^99^

While looking at the values listed in Table 5, it is clear that at RT the rate constant of the CN radical with cyanoethene is nearly 1 order of magnitude lower than those measured for the other two reactions. Unfortunately, there are no kinetic measurements at cryogenic temperatures.

To summarize, the reaction CN(X^2^Σ^+^) + C_2_H_3_CN appears to be a relatively simple system with only two channels open, leading mainly to *E*-but-2-enedinitrile for 65% and to its less thermodynamically stable isomer, *Z*-but-2-enedinitrile, for 35%. In both cases the reactive process culminates with the CN radical displacing one of the H atoms, increasing the molecular complexity. Interestingly, as our calculations show, the whole energetic profile connecting the intermediates, transition states and final products of channels **1a** and **1b** lies completely below the reactant asymptote. More importantly, the absence of an entrance barrier allows this reaction to occur efficiently even at extremely cold temperatures such as those found on Titan and in cold interstellar clouds.

If the key results of the present work, ideally complemented by future low temperature CRESU experiments, are implemented in the astrochemistry databases, modelers will be able to provide a more detailed description of the reactions involving the CN radical in extraterrestrial cold environments. Concerning cold clouds where both CN and C_2_H_3_CN have been observed with comparable abundances, we strongly suggest to search for *E*-but-2-enedinitrile and *Z*-but-2-enedinitrile which appear to be the best dinitrile candidates for future detection.

Regarding Titan’s atmosphere on the other hand, even though the reaction CN + C_2_H_3_CN is certainly not the main sink of the cyano radical, this reaction adds on to the possible chemical pathways (i) depleting the reservoir of cyanoethene, which is an ideal candidate to form alternative membrane-like structures in the apolar lakes of Titan, (ii) and contributing to build up toward the molecular complexity by incorporating a second CN group in the molecular skeleton of unsaturated hydrocarbons.

## Conclusions

In this study, we have investigated the reaction dynamics of CN + C_2_HCN from a theoretical and an experimental point of view. We have also performed RRKM and kinetics estimates at the conditions of the CMB experiment and at the low temperatures relevant to the ISM and Titan’s atmosphere. The key results can be summarized as follows:

- The PES reveals that, in order of decreasing exothermicity, there are four open reaction channels leading to (1) hydrogen atom and one of the three isomers of general formula C_4_H_2_N_2_, namely *E*-but-2-enedinitrile (*E*-NC–CH=CH–CN), *Z*-but-2-enedinitrile (*Z*-NC–CH=CH–CN), 1,1-dicyanoethene or methylene-propanedinitrile (CH_2_C(CN)_2_), or (2) cyanogen (NCCN) along with the H_2_CCH radical. These main reactive pathways lie below the reactant asymptote and have (except for the last case) no entrance barrier. Therefore, they are expected to be open also under the low temperature conditions of many extraterrestrial environments.
- The CMB data indicate the occurrence of two observable reactive channels with a relative ratio of 1 to 10. The major channel is consistent with the formation of H atom along with both the *E*- and *Z*-isomers of but-2-enedinitrile, but no further distinction between these two molecules can be inferred on the basis of the laboratory data alone. The minor channel is consistent with the formation of the 1,1-dicyanoethene and H atom resulting from an impulsive mechanism.
- RRKM and kinetics calculations based on a semiempirical model to treat the long-range interaction provide reliable rate coefficients and product branching ratios at the low temperatures typical of the atmosphere of Titan and cold interstellar clouds.

In conclusion, the title reaction is very likely to happen in both the ISM and on Titan, with a relatively fast kinetics, and hence we suggest to implement the information provided by our study in the databases employed by the astrochemistry community in order to improve the reliability and predictivity of their models. We also suggest that the *E*- and *Z*-isomers of but-2-enedinitrile as the best candidate for dinitriles detection in interstellar clouds.

## Supporting Information Available

Structures of the stationary points identified in the potential energy surface for the CN + C_2_H_3_CN reaction (PDF)
