# Supplementary material for: Unsaturated Dinitriles Formation Routes in Extraterrestrial Environments: A Combined Experimental and Theoretical Investigation of the Reaction between Cyano Radicals and Cyanoethene (C2H3CN)
Source: J Phys Chem A. 2022 May 31;126(22):3569–82. doi: 10.1021/acs.jpca.2c01802 (PMC9189926; doi:10.1021/acs.jpca.2c01802)

<u>**Electronic Supporting Information for the paper**</u>

Unsaturated Dinitriles Formation Routes in Extraterrestrial Environments: A Combined Experimental and Theoretical Investigation of the Reaction Between Cyano Radicals and Cyanoethene (C_2H_3CN)

Demian Marchione,[a] Luca Mancini,[a] Pengxiao Liang,[a] Gianmarco Vanuzzo,[a] Fernando Pirani[a], Dimitrios Skouteris,[b] Marzio Rosi,[c] Piergiorgio Casavecchia,[a] and Nadia Balucani [*,a]

a Dipartimento di Chimica, Biologia e Biotecnologie, Università degli studi di Perugia, 06123 Perugia, Italy

b MASTER TEC, 06123 Perugia, Italy

c Dipartimento di Ingegneria Civile ed Ambientale, Università degli studi di Perugia, 06123 Perugia, Italy

The following data report the cartesian coordinates for all the stationary points identified in the Potential Energy Surface, including minima, transition states, reactants and possible products, evaluated at the B3LYP/aug-cc-pVTZ level of theory. Together with the xyz cartesian coordinates of all the stationary points, the geometries are reported including the values of bond distances (in Å, in black) and angles (in °, in red).

S1: Structures of the stationary points identified in the PES for the C_2H_3CN + CN reaction

CN

C	0.00000	0.00000	-0.62586
N	0.00000	0.00000	0.53645

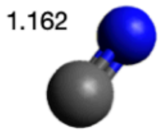

C_2H_3CN

C	1.60314	-0.35429	0.00000
C	0.58301	0.50089	0.00000
H	1.44874	-1.42395	0.00000
H	2.62185	0.00549	0.00000
C	-0.78167	0.08647	0.00000
H	0.74791	1.57100	0.00000
N	-1.89220	-0.22157	0.00000

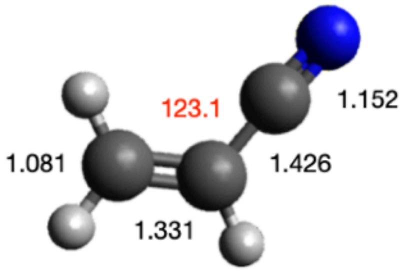

MIN1

C	0.51396	0.63520	-0.17035
C	-0.52891	-0.44270	-0.25952
C	-1.85661	-0.17625	0.00962
N	-2.97298	0.06042	0.24264
C	1.85529	0.10326	0.05404
N	2.90853	-0.32369	0.22219
H	0.27009	1.34107	0.62625
H	0.53067	1.21983	-1.09890
H	-0.25201	-1.43517	-0.58393

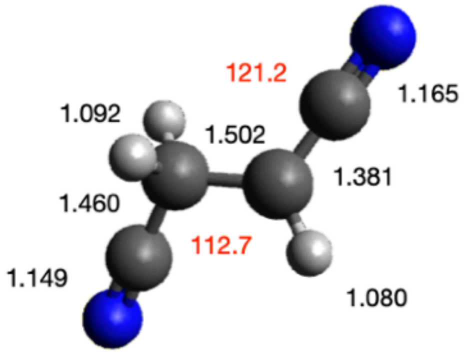

MIN2

C	0.77326	1.08412	0.00013
C	-0.72608	1.03799	-0.00015
C	-1.48134	-0.11422	0.00000
N	-2.14120	-1.07485	0.00007
H	-1.24830	1.98481	-0.00040
C	1.44295	-0.21162	-0.00002
H	1.11828	1.65111	0.87211
H	1.11859	1.65145	-0.87150
N	1.99243	-1.22015	-0.00007

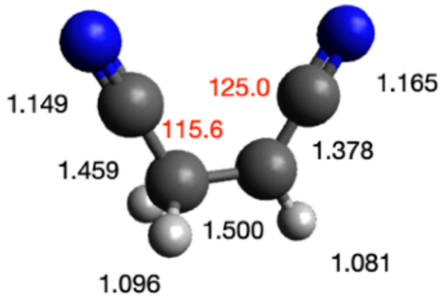

MIN3

C	1.70854	-1.03644	-0.00012
C	1.32380	0.23635	0.00017
H	0.99757	-1.85196	-0.00044
H	2.75785	-1.29460	-0.00003
C	-0.08830	0.66107	0.00000
H	2.03757	1.04877	0.00052
N	-0.43785	1.87484	-0.00013
C	-1.12968	-0.34702	-0.00002
N	-1.94489	-1.15855	0.00009

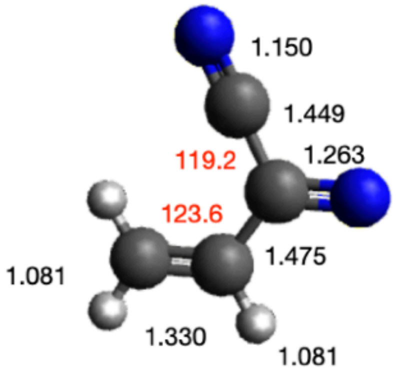

MIN4

C	-0.04904	1.74215	-0.21179
C	-0.00089	0.37959	0.42596
C	1.22974	-0.34779	0.08432
N	2.20948	-0.88604	-0.17769
C	-1.19514	-0.41182	0.06463.
N	-2.15481	-0.97902	-0.21369
H	0.45800	1.91850	-1.14610
H	-0.72890	2.47601	0.19036
H	-0.01985	0.48806	1.51660

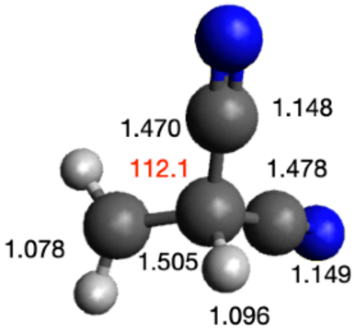

MIN5

C	-2.68891	0.54470	-0.01939
C	-1.72812	-0.43720	-0.18574
H	-2.43516	1.52932	0.34215
H	-3.71827	0.33555	-0.26163
C	-0.40052	-0.28398	0.00151
H	-2.01367	-1.41083	-0.57404
N	0.70532	-0.26876	0.53672.
C	1.89075	0.05463	0.01797
N	2.97009	0.30833	-0.30709

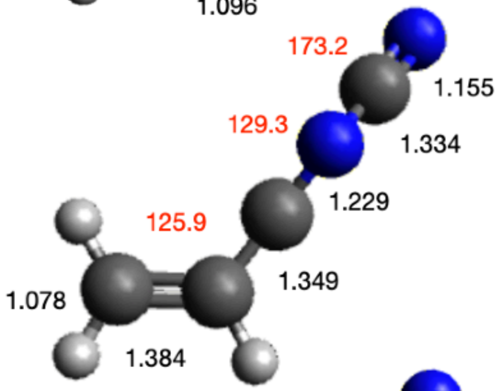

TS1

C	0.66007	-1.32553	-0.30408
C	-0.10184	-0.34801	0.55104
C	-1.39243	0.12837	0.09715
N	-2.42073	0.49082	-0.26613
C	1.09340	0.35294	0.08054
N	1.96976	1.10841	-0.16299
H	0.39271	-1.43921	-1.34127
H	1.29748	-2.04705	0.17897
H	-0.08868	-0.55502	1.61826

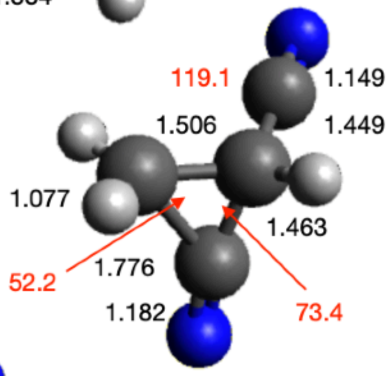

TS2

C	-0.47390	0.41878	0.15338
C	0.50694	-0.45624	-0.16250
C	1.87891	-0.14099	-0.00923
N	3.00210	0.09451	0.10670
C	-1.85087	0.05849	0.08176
N	-2.96720	-0.22285	0.04591
H	-0.63882	1.68451	-1.50442
H	-0.24385	1.36421	0.62556
H	0.27176	-1.43052	-0.56980

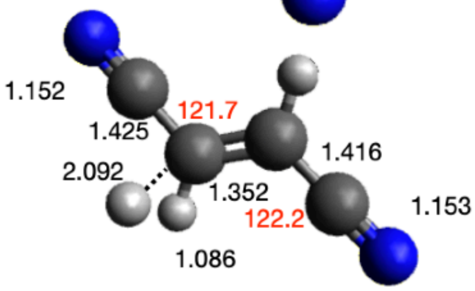

TS3

C	2.13291	-0.32606	0.00000
C	1.38681	0.73849	0.00000
H	1.72692	-1.33225	-0.00000
H	3.21935	-0.24117	0.00000
C	-0.92081	0.60574	0.00000
H	1.50012	1.80892	0.00000
N	-1.34564	1.69520	0.00000
C	-0.96690	-0.78025	0.00000
N	-0.97413	-1.93278	0.00000

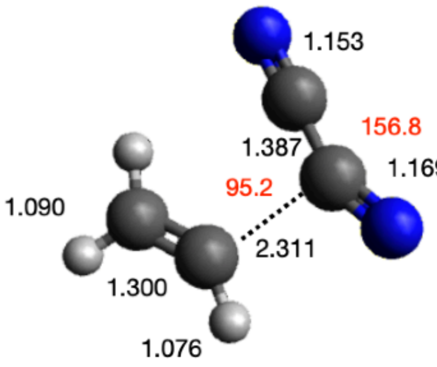

TS4

C	0.70486	1.00889	0.28384
C	-0.67956	0.93731	-0.30279
C	-1.54413	-0.11161	-0.05665
N	-2.28092	-0.98845	0.15287
C	1.51658	-0.18266	0.03361
N	2.16293	-1.11144	-0.16520
H	0.64711	1.15546	1.36853
H	1.22805	1.87437	-0.12208
H	-1.03576	1.75786	-0.90821

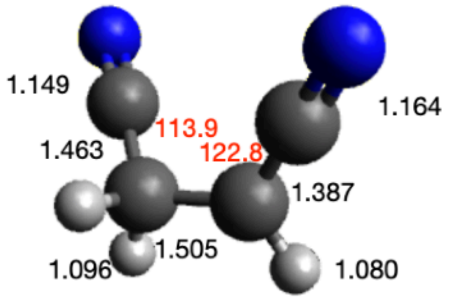

TS5

C	1.25351	-1.48294	0.00012
C	1.56890	-0.19079	-0.00016
H	0.22853	-1.82397	0.00054
H	2.03603	-2.22762	-0.00007
C	0.63865	0.89462	0.00001
H	2.60179	0.13673	-0.00057
N	0.13244	1.94666	0.00000
C	-1.52668	-0.03606	0.00049
N	-2.48566	-0.68868	-0.00038

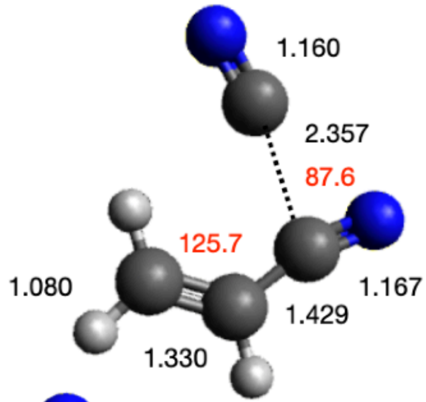

TS6

C	-0.67218	0.98688	-0.10089
C	0.67942	1.01094	-0.13623
C	1.50716	-0.13012	-0.00770
N	2.21751	-1.03326	0.09240
H	1.18674	1.96004	-0.24471
C	-1.44743	-0.20765	-0.05131
H	-1.22069	1.90160	-0.27754
H	-1.16033	1.48389	1.87131
N	-2.10431	-1.15329	-0.03130

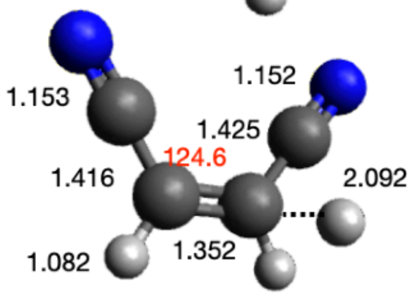

TS7

C	-0.89895	-1.23377	-0.06103
C	0.54980	-1.26389	0.06591
C	0.81567	0.14464	-0.07600
N	1.84435	0.87773	-0.01565
H	1.20460	-2.03822	0.43226
C	-0.64435	0.53349	-0.02839
N	-1.45897	1.40624	0.09970
H	-1.52560	-1.77357	0.64596
H	-1.30968	-1.25880	-1.06948

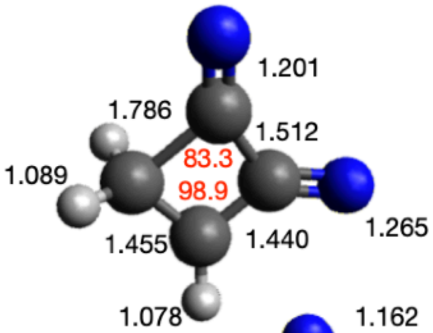

TS8

C	0.77696	1.64089	-0.33228
C	1.36121	0.67853	0.38149
C	1.28480	-0.70288	0.03225
N	1.22275	-1.82619	-0.22063
C	-1.87519	-0.33256	0.15041
N	-2.97759	0.01051	0.01920
H	0.22062	1.42561	-1.23334
H	0.85113	2.67388	-0.02490
H	1.92539	0.90633	1.27705

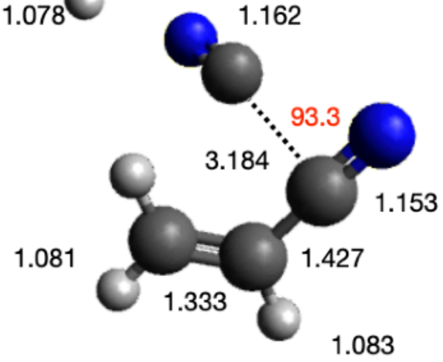

TS9

C	0.00002	1.73992	-0.10645
C	0.00001	0.39346	0.08233
C	1.22604	-0.35808	-0.00812
N	2.21133	-0.94416	-0.10030
C	-1.22602	-0.35808	-0.00812
N	-2.21130	-0.94416	-0.10036
H	0.92975	2.28508	-0.15948
H	-0.92970	2.28510	-0.15945
H	-0.00049	0.14479	1.96570

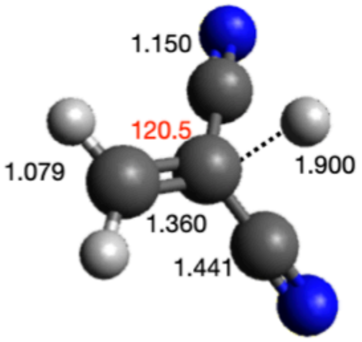

TS10

C	2.29210	-0.72923	0.00000
C	1.55043	0.37967	-0.00001
H	1.83302	-1.70823	0.00002
H	3.37157	-0.67949	-0.00001
C	0.11687	0.33601	0.00000
H	1.99686	1.37124	-0.00003
N	-0.84728	1.16665	0.00001
C	-1.49321	-0.07495	0.00000
N	-2.29537	-0.94558	-0.00001

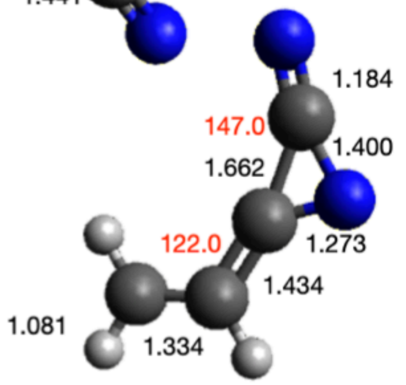

E-NC-CH=CH-CN

C	-0.48638	0.46058	-0.00038
C	0.48638	-0.46058	-0.00038
C	1.86375	-0.11174	0.00008
N	2.98774	0.14250	0.00034
C	-1.86375	0.11174	0.00008
N	-2.98775	-0.14250	0.00034
H	-0.25467	1.51772	-0.00060
H	0.25467	-1.51772	-0.00060

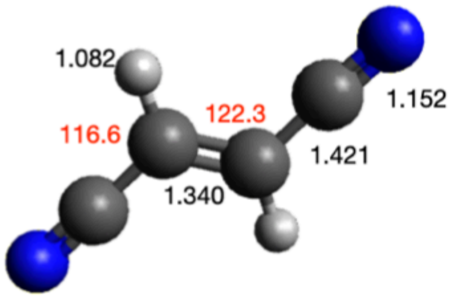

Z-NC-CH=CH-CN

C	0.67014	1.03479	0.00167
C	-0.66998	1.03475	-0.00168
C	-1.47665	-0.13457	-0.00130
N	-2.17125	-1.05395	0.00152
C	1.47683	-0.13450	0.00115
N	2.17096	-1.05422	-0.00143
H	-1.20131	1.97713	-0.00390
H	1.20139	1.97725	0.00418

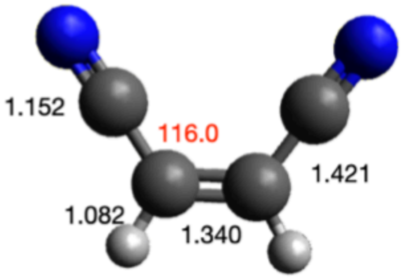

$CH_2C(CN)_2$

C	-0.00227	1.74281	-0.00013
C	0.00010	0.40416	0.00028
C	-1.21981	-0.34986	0.00004
N	-2.20166	-0.95007	-0.00010
C	1.22199	-0.34655	0.00010
N	2.20257	-0.94867	-0.00012
H	-0.93159	2.29238	-0.00005
H	0.92520	2.29549	-0.00016

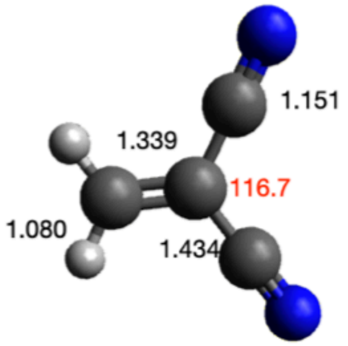

TS-iso

C	0.71075	0.59437	-0.60697
C	-0.44830	0.77891	0.24185
H	1.32055	1.47412	-0.75798
H	0.53630	0.05404	-1.52705
H	-0.41026	1.49892	1.04709
C	-1.59777	0.01274	0.10348
N	-2.57041	-0.61160	-0.01896
N	1.78107	-0.94502	-0.13040
C	2.01511	-0.07448	0.64223

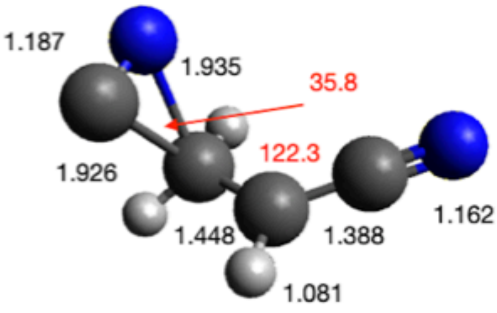

NCCN

C	0.00000	0.00000	0.68786
N	0.00000	0.00000	1.84006
C	0.00000	0.00000	-0.68786
N	0.00000	0.00000	-1.84006

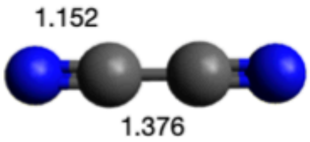

CH_2CH

C	0.58557	0.02928	0.00007
C	-0.70447	-0.14161	-0.00004
H	1.03472	1.02353	-0.00016
H	1.28054	-0.80503	-0.00011
H	-1.60186	0.45547	0.00007

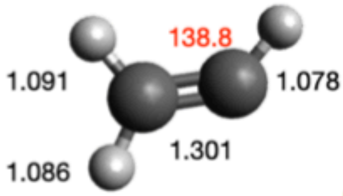

$CH_2C(N)CCN$

C	2.28621	-0.68738	-0.00001
C	1.14857	-0.03103	0.00002
H	2.29687	-1.76648	-0.00000
H	3.22587	-0.15497	-0.00005
C	-0.22862	0.22394	0.00001
N	0.51526	1.26893	-0.00001
C	-1.58944	-0.12326	0.00001
N	-2.68999	-0.46495	-0.00001

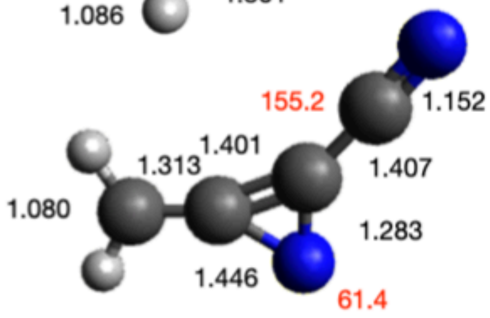

HCCN

C	-0.06935	-1.27358	0.00000
C	0.00000	0.09212	0.00000
N	0.21709	1.24600	0.00000
H	-1.10348	-1.63326	0.00000

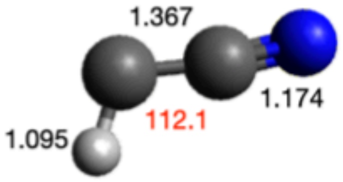

CH_2CN

C	0.00024	-1.18907	0.00000
C	0.00000	0.18727	0.00000
N	-0.00027	1.35325	0.00000
H	0.00025	-1.73099	0.93324
H	0.00025	-1.73099	-0.93324

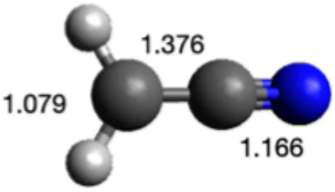

S2: Structures of the stationary points identified in the PES for the C_2H_3CN + NC reaction

MIN6

C	-0.85682	1.01071	0.00019
C	0.64033	1.04832	-0.00020
C	1.45362	-0.06261	-0.00012
N	2.16170	-0.98822	0.00016
N	-1.40992	-0.29241	0.00000
C	-1.88765	-1.35678	-0.00014
H	-1.23229	1.55080	0.87599
H	-1.23273	1.55126	-0.87512
H	1.10572	2.02452	-0.00046

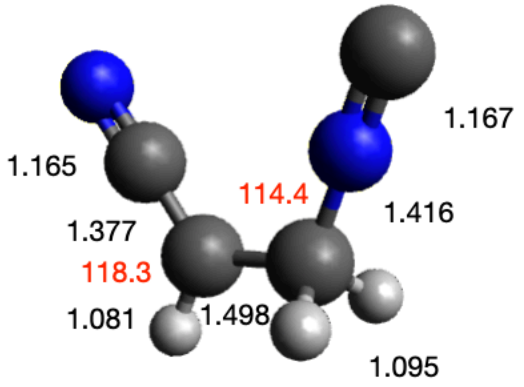

MIN7

C	0.11524	1.72171	-0.20897
C	0.05743	0.35747	0.40759
H	-0.78622	2.30884	-0.27340
H	1.07355	2.13727	-0.47302
C	-1.19602	-0.33959	0.05800
N	-2.19592	-0.84434	-0.19412
N	1.18974	-0.43738	0.05386
C	2.13880	-1.05932	-0.21933
H	0.06335	0.44427	1.50456

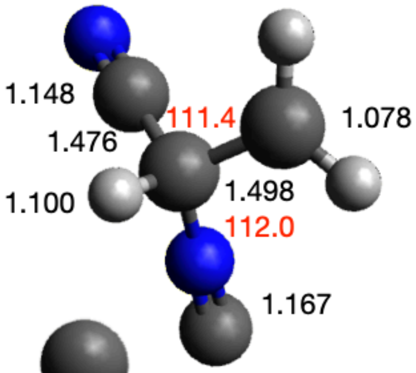

MIN8

C	-0.54935	0.61503	0.12608
C	0.48930	-0.46591	0.17410
C	1.82679	-0.17550	-0.00771
N	2.95098	0.08409	-0.16594
N	-1.85658	0.08759	-0.04250
C	-2.93084	-0.34601	-0.17706
H	-0.33845	1.31352	-0.68663
H	-0.53013	1.19866	1.05354
H	0.19242	-1.47964	0.39974

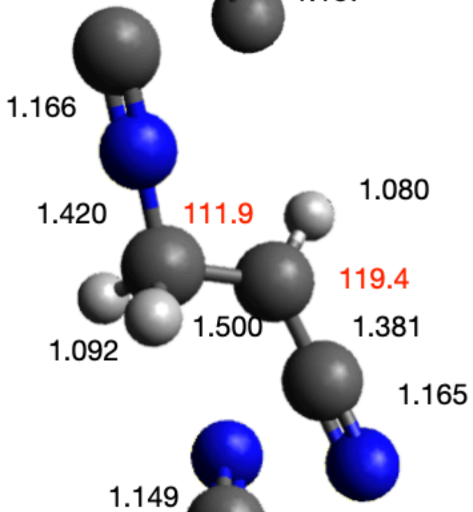

TS12

C	-0.76796	1.26030	-0.29956
C	0.05870	0.33529	0.55644
C	1.36469	-0.08963	0.09952
N	2.40678	-0.41384	-0.26133
N	-1.08783	-0.34203	0.02136
C	-1.87696	-1.26643	-0.15910
H	-0.47360	1.42373	-1.32241
H	-1.44779	1.93825	0.18854
H	0.01791	0.49193	1.62986

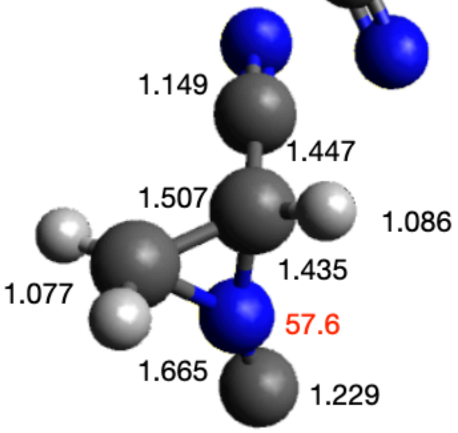

TS13

C	-0.51734	0.40269	-0.13548
C	0.46786	-0.47751	0.15305
C	1.83276	-0.13995	0.00449
N	2.95228	0.11461	-0.10898
N	-1.83943	0.04378	-0.07338
C	-2.98064	-0.21976	-0.04924
H	-0.31529	1.35589	-0.60326
H	-0.52284	1.60355	1.50664
H	0.23244	-1.46092	0.53625

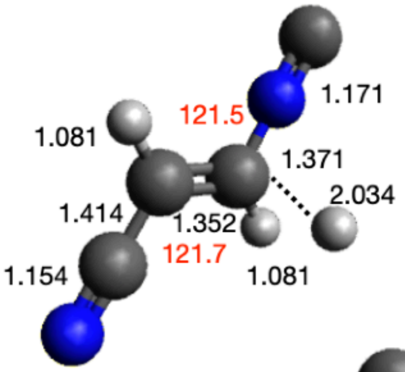

TS14

C	0.13674	1.72165	-0.11892
C	0.06230	0.38240	0.08513
H	-0.76446	2.31278	-0.15086
H	1.09586	2.21050	-0.18741
C	-1.19809	-0.31391	-0.00620
N	-2.20968	-0.85265	-0.10074
N	1.19338	-0.41777	-0.00910
C	2.13678	-1.10400	-0.10027
H	-0.04380	0.25278	1.94870

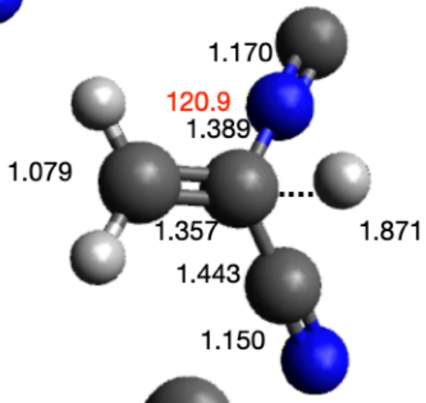

TS15

C	0.74840	0.91610	-0.09745
C	-0.60065	1.01080	-0.13625
C	-1.48042	-0.08741	-0.00919
N	-2.22738	-0.96125	0.08787
N	1.41764	-0.27764	-0.04720
C	2.03415	-1.27390	-0.01724
H	1.36360	1.78217	-0.29360
H	1.14110	1.50618	1.80881
H	-1.04533	1.99047	-0.23921

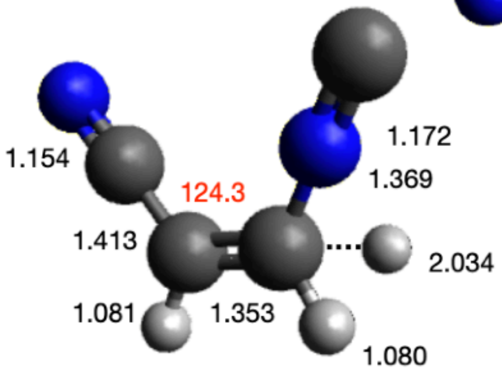

E-NC-CH=CH-NC

C	-0.52624	0.43323	-0.00006
C	0.45068	-0.48066	-0.00006
C	1.82000	-0.10828	-0.00004
N	2.93973	0.16505	0.00008
N	-1.84984	0.09213	0.00001
C	-2.99957	-0.13721	0.00006
H	-0.32123	1.49527	-0.00009
H	0.22274	-1.53805	-0.00003

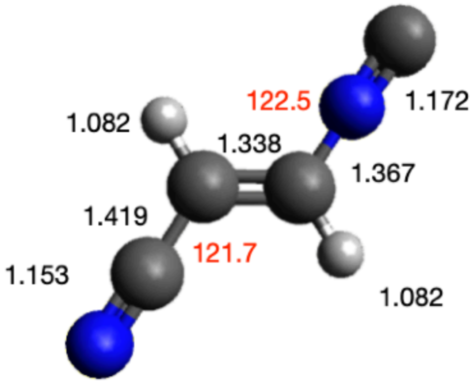

Z-NC-CH=CH-NC

C	0.74422	0.96647	0.00071
C	-0.59250	1.03399	-0.00061
C	-1.45344	-0.09337	-0.00093
N	-2.18540	-0.98342	0.00086
N	1.45325	-0.20021	0.00134
C	2.10936	-1.17213	-0.00147
H	1.34120	1.86789	0.00127
H	-1.06196	2.00768	-0.00285

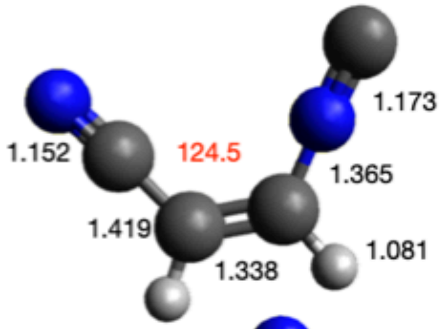

CH_2CCNNC

C	-0.13020	1.72720	0.00000
C	-0.06264	0.39449	0.00000
H	0.77392	2.31532	0.00000
H	-1.08483	2.23055	0.00000
C	1.19099	-0.30466	0.00000
N	2.20149	-0.85492	0.00000
N	-1.18966	-0.40397	0.00000
C	-2.12680	-1.10597	0.00000

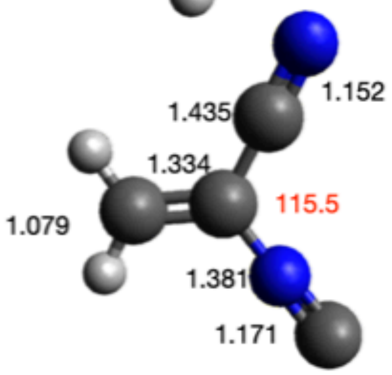

TS-Abs

C	0.01584	0.69996	-0.00000
C	0.84068	-0.33535	-0.00002
H	0.27806	1.74940	0.00002
H	-1.12786	0.49196	-0.00002
C	2.25839	-0.17525	0.00000
H	0.47012	-1.35330	-0.00004
N	3.40539	-0.06715	0.00002
C	-2.64857	0.07190	-0.00003
N	-3.75088	-0.28365	0.00003

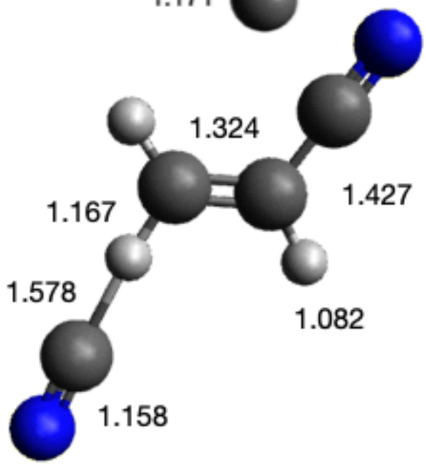

TS-Mig

C	0.45343	0.43810	-0.07283
C	-0.55301	-0.50154	0.01560
C	-1.90776	-0.12789	-0.00052
N	-3.02669	0.16540	-0.02342
C	1.85042	0.12868	0.01801
N	2.98349	-0.18463	-0.09185
H	0.22622	1.48301	-0.23939
H	1.33942	0.57973	1.16398
H	-0.32179	-1.55219	0.12067

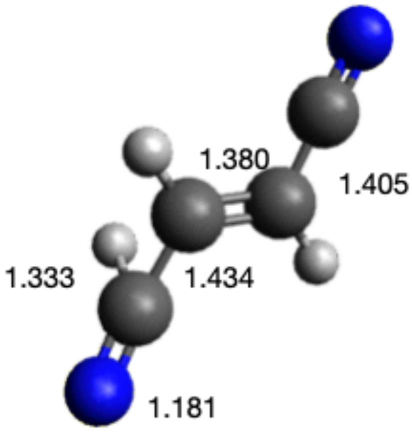

Supplement: Supplementary file 1 — jp2c01802_si_001.pdf [file jp2c01802_si_001.pdf]
